# Evaluating Rational Drug Use in Developing Countries: A Cross‐Sectional Analysis of Prescribing Patterns Against WHO Indicators in Pakistan and Yemen

**DOI:** 10.1002/prp2.70174

**Published:** 2025-11-19

**Authors:** Amjad Khan, Ali Mohammed Ahmed Al‐Subari, Saima Mushtaq, Kiran Hameed, Sameen Abbas, Muhammad Iqbal, Yalin Dong, Weiyi Feng, Yu Fang

**Affiliations:** ^1^ Department of Pharmacy The First Affiliated Hospital of Xi'an Jiaotong University Xi'an China; ^2^ Department of Pharmacy Administration and Clinical Pharmacy, School of Pharmacy, Health Science Center Xi'an Jiaotong University Xi'an China; ^3^ Department of Pharmacy Quaid‐i‐Azam University Islamabad Pakistan; ^4^ Faculty of Pharmacy Gomal University Dera Ismail Khan Pakistan

**Keywords:** antibiotic misuse, drug use evaluation, Pakistan and Yemen healthcare, polypharmacy, rational drug use, WHO prescribing indicators

## Abstract

The rational use of medications is essential for optimal healthcare delivery, especially in developing countries. This research delves into prescribing patterns within the healthcare system of Pakistan and Yemen against World Health Organization (WHO) prescribing indicators to uncover variances from recommended practices. A cross‐sectional study was conducted in the outpatient department of different hospitals in Pakistan and Yemen, including 400 prescriptions. Demographic details and prescription data were collected, focusing on WHO prescribing indicators including the average number of drugs per encounter, the percentage of drugs prescribed by generic name, the percentage of drugs prescribed from the essential drug list, and the prevalence of antibiotic and injectable prescriptions. The mean age of patients was 43.54 ± 16.92 and 38.81 ± 18.67 years in Pakistan and Yemen, respectively. Polypharmacy was observed in both populations, with an average of 5.44 ± 1.16 drugs per encounter in Pakistan and 6.18 ± 1.09 in Yemen. Irrational antibiotic use was observed, with rates of 65% and 72.5% encounters in Pakistan and Yemen, respectively. The mean injectable medications per hospital were 28.5 (57%) and 34 (68%) in Pakistan and Yemen, respectively. In Pakistan, the mean percentage of generic prescribing was 74.18%, while in Yemen, it was 78.24%. The total percentage of drugs prescribed from the essential drug list was 98.24% in Pakistan, whereas 97.87% in Yemen. The findings reveal significant deviations from WHO prescribing indicators marked by polypharmacy and excessive antimicrobial and injectable utilization, which pose risks to patient safety and contribute to antimicrobial resistance.

## Graphical abstract

An Assessment of Prescribing Patterns Against WHO Indicators for Rational Medicine Use in Pakistan and Yemen.

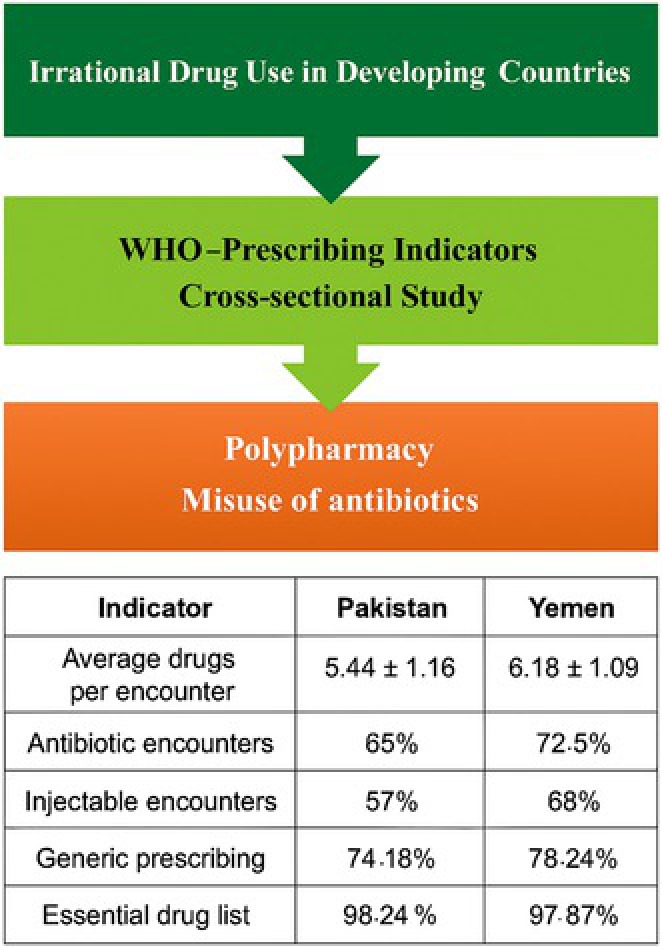

## 1 Introduction

Medicines are integral to the healthcare system and are pivotal in effectively managing various health conditions. Rational drug use refers to administering drugs according to specific clinical requirements, in doses specified for individual patients' needs, for an appropriate duration, and at minimum cost to both patient and community [1]. When prescribed rationally, they can significantly enhance patient outcomes and quality of life (QoL); however, irrational use of medication results in prolonged illness, adverse drug reactions, and unnecessary economic burden. Proper administration of pharmaceutical drugs plays a vital role in the healthcare system for effective patient treatment. Drug use evaluation helps to find actual practices and identify problems to promote rational drug use, cost‐effectiveness, and improved patient outcomes. Irrational drug use is highly prevalent worldwide and involves inappropriate antibiotic utilization, polypharmacy, and incorrect drug administration [2]. The World Health Organization (WHO) emphasizes the importance of drug use evaluation that promotes irrational prescribing practices, particularly in developing countries like Pakistan and Yemen [3]. In developing countries, 20 to 50% of the healthcare budget is spent on medicines and other health supplies, where irrational drug use results in waste of resources and a lack of confidence in healthcare systems [4]. Studies highlight the prevalence of irrational prescribing practices in both populations. The average number of drugs per prescription is 4.4 in Pakistan, that is, one of the highest worldwide. Misuse of antibiotics is prevalent, with approximately 70% of patients utilizing inappropriate antibiotics [5]. There is also a serious concern that 31% of drug dosages are found to be incorrect, and 60% of patients are prescribed unnecessary injectables. More than 50% of drugs are prescribed by brand names, leading to exacerbating economic strains on patients. Similarly, in Yemen, the percentage of antibiotic prescribing was reported as 84.2%, leading to antibiotic resistance in the region [6]. Another study found that around 63.54% of admitted patients were prescribed two or more antibiotics [7]. Moreover, a study reported unnecessary injectable drug use in almost 33% of all prescribed drugs [6]. This widespread irrational prescribing in both populations not only increases the risk of adverse drug reactions but also contributes to the growing threat of antimicrobial resistance (AMR), leading to high economic burdens.

The healthcare system of Yemen has been under considerable strain since the escalation of conflict in 2015 [8]. WHO estimates that around 50% of public health facilities in Yemen are not fully operational, with a shortage of personnel and a lack of essential drugs and equipment [9]. Therefore, prescribing practices have also fragmented under these constraints, with a substantial prevalence of irrational prescribing. Similarly, Pakistan is also struggling with multiple challenges, including the prevalence of infectious diseases, aggravated by a persistent scarcity of essential drugs and unclear health and drug policies. These issues hinder the effective medication management process, creating an environment for healthcare professionals to practice irrational prescribing that significantly impacts the healthcare system [10]. Polypharmacy is a common practice in both countries and a leading cause of irrational prescribing, resulting in harmful drug–drug interactions. Excessive drug use increases the risks of treatment complications and adverse drug reactions, particularly in hospitalized and elderly populations [11, 12].

In light of these challenges, it is crucial to determine actual prescribing patterns in healthcare settings against WHO prescribing indicators in Yemen and Pakistan. Therefore, the study aimed to identify deviations from recommended practices and develop targeted interventions to promote rational prescribing and improve patient outcomes in both countries.

## 2 Methodology

### 2.1 Study Participants and Recruitment

This study employed a cross‐sectional design to identify the prescribing patterns in outpatient departments across the selected hospitals in Yemen and Pakistan. Four hundred prescriptions (200 from each country) were analyzed from eight different hospitals, four in Yemen and four in Pakistan. A systematic random sampling technique was used to select 50 prescriptions from each hospital in both countries, from November 2023 to May 2024.

The inclusion criteria included all prescriptions containing drugs issued during the study duration, while incomplete prescriptions (missing dosage, frequency, or drug name), illegible, duplicate, or prescriptions containing solely medical equipment and supplies were excluded. Missing prescriptions were identified by comparing registration logs with available prescription records, and any untraceable entries were excluded from the study. Informed consent was obtained from each hospital's administration.

### 2.2 Data Collection

Outpatient prescriptions were captured by well‐trained pharmacists using cameras, which ensured that all data were recorded accurately. Demographic details, signs and symptoms data from clinical examinations, and medication details were collected. The prescriptions were analyzed using WHO guidelines to evaluate drug use practices in healthcare settings. Data was recorded using the WHO prescribing indicators form. The study utilized standardized WHO prescribing indicators, including the average number of drugs per encounter, percentage of medications prescribed by generic name, percentage of drugs prescribed from the essential drug list, and percentage of encounters at which antibiotics were prescribed [10]. Data was analyzed using the Statistical Package for Social Sciences (SPSS) version 20. Descriptive statistics were used to analyze data, including average/mean, frequencies, and percentages.

### 2.3 Operational Definitions

#### 2.3.1 Average Number of Drugs per Encounter

The average number of drugs prescribed per encounter was calculated to assess the degree of polypharmacy. It was calculated using the following formula:

$$\text{Average}\;\mathrm{no}.\;\text{of drugs}\;\mathrm{per}\;\text{encounter}=\frac{\text{Total}\;\mathrm{no}.\;\text{of drugs prescribed}}{\text{Number of encounters}}$$

#### 2.3.2 Percentage of Drugs Prescribed by Generic Name

The percentage of drugs prescribed under generic names was assessed using the following formula to measure the tendency of generic prescribing practices.

$$\begin{array}{c} \%\text{of drugs prescribed}\;\mathrm{by}\;\text{generic names}\\ \;=\frac{\text{Drugs prescribed}\;\mathrm{by}\;\text{generic names}}{\text{Total drugs prescribed}}\times 100 \end{array}$$

#### 2.3.3 Percentage of Encounters With an Antibiotic‐Prescribed

To measure the overutilization and misuse of antibiotics, the percentage of encounters per prescription was calculated as

$$\begin{array}{c} \%\text{of drugs prescribed with antibiotics}\\ \;=\frac{\mathrm{No}.\;\text{of patients encounters with antibiotic}}{\text{Total}\;\mathrm{no}.\;\text{of encounters}}\times 100 \end{array}$$

#### 2.3.4 Percentage of Encounters With an Injection Prescribed

The percentage of encounters prescribing injections was calculated using the following formula to find the overutilization of injectables.

$$\begin{array}{c} \%\text{of drugs prescribed with injection}\\ \;=\frac{\mathrm{No}.\;\text{of patients encounters with injection}}{\text{Total}\;\mathrm{no}.\;\text{of encounters}}\times 100 \end{array}$$

#### 2.3.5 Percentage of Drugs Prescribed From the Essential Drug List

The percentage of drugs prescribed from the National Essential Medicine List (NEML) was calculated as

$$\begin{array}{c} \%\text{of drugs prescribed from}\;\mathrm{EDL}\\ \;=\frac{\mathrm{No}.\;\text{of patients encounters from}\;\mathrm{EDL}}{\text{Total}\;\mathrm{no}.\;\text{of drugs priscribed}}\times 100 \end{array}$$

## 3 Results

Prescriptions of 400 outpatients from both countries were investigated. Patients were categorized into different age groups, with the mean age found to be 43.54 ± 16.92 years in Pakistan and 38.81 ± 18.67 years in Yemen. Among the study participants, 54.5% were males, 45.5% were females from Pakistan, 65.5% were males, and 34.5% were females from Yemen. The demographic data of the patients is presented below (Table 1).

**TABLE 1:** Sociodemographic characteristics of study populations (*n* = 400).

Characteristics	Pakistan		Yemen	
	Frequency	Percentage	Frequency	Percentage
*Age (Years)*				
11–20	2	1	14	7
21–30	42	21	51	25.5
31–40	57	28.5	40	20
41–50	29	14.5	28	14
51–60	26	13	27	14.5
61 and above	39	19.5	31	15.5
Average patient's age (years), (SD)	43.54 ± 16.92		38.81 ± 18.67	
*Gender*				
Male	109	54.5	131	65.5
Female	91	45.5	69	34.5
*Educational qualifications*				
No formal education	23	11.5	22	11
Primary education	22	11	27	13.5
Secondary education	36	18	55	27.5
Tertiary or post‐secondary education	119	59.5	94	47
*Marital status*				
Single	34	17	56	28
Married	148	74	126	63
Divorced	5	2.5	6	3
Widowed	13	6.5	12	6
*Occupation*				
Trading/business	20	10	19	9.5
Public/civil servant	92	46	64	32
Artisans	35	17.5	44	22
Student	30	15	53	26.5
Farming	23	11.5	20	10

Abbreviations: *N*, number; SD, standard deviation.

### 3.1 Average Number of Drugs per Encounter

The average number of drugs prescribed per prescription from four hospitals in Pakistan and Yemen was calculated (Table 2). In Pakistan, 1088 drugs were prescribed, with an average of 272 per hospital, which was 5.44 ± 1.16. In Yemen, 1236 drugs were prescribed, with an average of 309 per hospital. The average number of drugs per encounter was 6.18 ± 1.09.

**TABLE 2:** Comparison of average drugs per encounter in Pakistan and Yemen government hospitals.

Government hospital	No. of prescriptions encounter per hospital	Average No. of drugs per encounter (*N*; Mean ± SD)	
		Pakistan	Yemen
G‐1	50	250; 5.00 ± 1.07	276; 5.52 ± 0.81
G‐2	50	263; 5.26 ± 1.17	298; 5.96 ± 1.34
G‐3	50	295; 5.90 ± 1.28	330; 6.60 ± 1.16
G‐4	50	280; 5.60 ± 1.24	332; 6.64 ± 1.08
Total	200	1088	1236
Mean		272; 5.44 ± 1.16	309; 6.18 ± 1.09

Abbreviations: G, government; *N*, number; SD, standard deviation.

### 3.2 Percentage of Drug Encounters With an Antibiotic

The percentage of drug encounters with an antibiotic in Pakistan and Yemen is presented below (Table 3). Among 200 prescriptions, 130 in Pakistan and 145 in Yemen have antibiotics. The mean percentage of medicines encountered with an antibiotic was 65% and 72.50% in Pakistan and Yemen, respectively.

**TABLE 3:** Comparison of drug encounters percentage with an antibiotic in government hospitals of Pakistan and Yemen.

Government hospital	Number of prescriptions encounter per hospital	Percentage of drug encounters with an antibiotic *n* (%)	
		Pakistan	Yemen
G‐1	50	34 (68)	26 (52)
G‐2	50	38 (76)	36 (72)
G‐3	50	31 (62)	41 (82)
G‐4	50	27 (54)	42 (84)
Total	200	130 (65)	145 (72.5)
Mean		32.5 (65)	36.25 (72.50)

Abbreviations: G, government; *N*, number.

### 3.3 Comparison of Drug Encounter Percentage With an Injection

The mean values of the drug encounter percentage with an injection in government hospitals in Pakistan and Yemen are presented below (Table 4). Among the 200 prescriptions, injectable drugs were prescribed in 57% of Pakistani prescriptions, while in Yemen, they were prescribed in 68% of Yemeni prescriptions. Similarly, the mean percentage of injection prescriptions per hospital was 28.5 (57%) and 34 (68%) in Pakistan and Yemen, respectively.

**TABLE 4:** Comparison of drug encounters percentage with an injection in government hospitals of Pakistan and Yemen.

Government hospital	Number of prescriptions encounter per hospital	Percentage of drug encounters with an injection *n* (%)	
		Pakistan	Yemen
G‐1	50	27 (54)	31 (62)
G‐2	50	30 (60)	34 (68)
G‐3	50	28 (56)	33 (66)
G‐4	50	29 (58)	38 (76)
Total	200	114 (57)	136 (68)
Mean		28.5 (57)	34 (68)

Abbreviations: G, government; *N*, number.

### 3.4 Comparison of Total Drugs Prescribed in Generic

**FIGURE 1:**
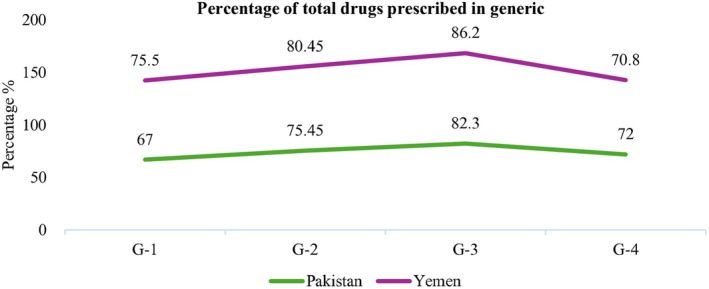
Comparison of generic drug prescriptions in government hospitals of Pakistan and Yemen.

The total drug percentage prescribed in generic form in the government hospitals of Pakistan and Yemen is shown below (Figure 1). In Pakistan, the mean percentage of total drugs prescribed in generic form was 74.18%, while in Yemen, it was 78.24%.

### 3.5 Comparison of Total Drugs Prescribed From the Essential Drug List

The percentage of total drugs prescribed from the essential drug list in the government hospitals of Pakistan and Yemen was investigated in the present study (Table 5). The percentage of drugs prescribed from the essential drug list in Pakistan was 98.24%, whereas in Yemen, it was 97.87%.

**TABLE 5:** Comparison of total drugs prescribed from EDL in government hospitals of Pakistan and Yemen.

Government hospital	Number of prescriptions encounter per hospital	Percentage of total drugs prescribed from EDL	
		Pakistan	Yemen
G‐1	50	98.00	96.00
G‐2	50	97.65	98.50
G‐3	50	99.00	98.00
G‐4	50	98.30	99.00
Mean	—	98.24	97.87

Abbreviations: EDL, essential drug list; G, government.

## 4 Discussion

The evaluation of prescribing patterns against WHO indicators is imperative to identify the practices of rational drug use, especially in developing countries like Pakistan and Yemen. The current study aimed to assess prescribing patterns and ascertain deviations from established guidelines, contributing to the existing body of literature on drug utilization. The study findings highlighted substantial concerns related to prescribing practices in both countries. This study found that the average number of drugs per encounter was 5.44 and 6.18 in Pakistan and Yemen, respectively, both exceeding the WHO recommended limit of 1.6–1.8 [13]. These findings are higher than a study from Pakistan, which reported an average of 3.1 drugs per encounter [14], whereas another study in Yemen found the average to be three [15]. The high index of polypharmacy in the current study aligns with the previous research, indicating widespread use of irrational prescribing in these regions ^511^. This pattern of polypharmacy raises significant concerns about the risk of adverse drug reactions and potential drug interactions associated with high rates of hospitalization and mortality, especially in vulnerable populations such as the elderly. In developing countries like Yemen and Pakistan, where healthcare resources are limited, managing multiple medications becomes challenging for patients, leading to non‐adherence to the therapy and increased healthcare expenditures. Moreover, limited consultation time, lack of diagnostic services, prescribers practices of empirical treatment, and patients expectations and demands for quick relief are the factors contributing to the high rate of polypharmacy.

Moreover, this study also revealed that antibiotics were prescribed in 65% of encounters in Pakistan, which is lower than another study indicating a rate of 76.4% [10]. In Yemen, antibiotic prescribing was found to be 72.4% less than 84.2% reported in another study [6]. This excessive reliance on antibiotics not only challenges patient safety but also contributes to global crises of AMR, highlighting the urgent need for strategic measures to curb irrational antibiotic prescribing. This emphasizes the immediate need for a comprehensive antimicrobial stewardship program that advises healthcare providers on appropriate prescribing practices [3].

The current study found that the percentage of generic prescribing was 74.18% in Pakistan, which is considerably higher than previous studies showing 0.11% and 4.8% [14, 16]. The percentage of drugs prescribed by generic name was found to be 78.24% in Yemen, which is significantly higher than 24.29% found by another study [17]. The findings were close to the WHO WHO‐recommended limit of 100% [13].

The analysis further revealed that the average injectable encounters per prescription were 57% in Pakistan and 68% in Yemen. These counts exceed the WHO's recommended range, which is 13.4% to 24.1% [18]. The over‐prescribing of injectables is a common trend observed in other studies from Yemen [15, 18]. The overuse of injectables is associated with a high risk of infections and increased costs for patients. These results underscore the need for healthcare providers to adhere to the recommended guidelines that promote oral medication prescribing over injectables when appropriate. Moreover, the higher rates of injectable prescribing might be related to the physical need for rapid effect, or in the case of critically ill patients who are unable to take oral medication, can be prescribed with parenteral dosage forms. However, the unjustifiable prescribing of injectables should be discouraged to promote rational drug use.

The percentage of medications prescribed from the essential drug list (EDL) was relatively close to WHO recommended limits in both countries, with Yemen at 97.87% and Pakistan at 98.24%. These figures suggest positive adherence to essential drug prescribing practices in contrast to previous studies that reported inconsistency in prescribing from the EDL in Pakistan [18] and comparable to a study from Yemen [19]. The high adherence to EDL prescribing in both countries reflects alignment of their National Essential Drug Lists with the WHO model lists of essential medicines.

Additionally, current studies need to explore the pharmacist's role in influencing prescribing practices despite their pivotal role in medication management. This finding is in contrast to other studies that highlight that pharmacists can promote rational prescribing through patient education and collaboration with prescribers [20].

The results of the current study have significant outcomes for healthcare policymakers and practitioners. The emergence of polypharmacy and the lack of rational antibiotic prescribing underline the pressing need to implement educational programs aimed at healthcare providers. Furthermore, achieving this goal requires the establishment of stringent regulatory frameworks to encourage compliance with the WHO's guidelines.

In Pakistan, interventions such as prescribers' education, regular drug audits, implementation of national standard treatment guidelines, and adoption of electronic prescription systems are necessary to promote rational drug use. In Yemen, international cooperation is essential to strengthen medicine supply chains and rebuild regulatory frameworks disrupted by conflicts. Both countries should focus on antibiotic stewardship programs and patient education initiatives to address irrational drug use.

## 5 Limitations

The study has several limitations, employing the cross‐sectional approach, which limits the ability to establish causal correlation between prescribing behaviors and outcomes. The study duration was limited; that is not adequate to represent drug utilization variation over time. Reliance on prescription data only may overlook the key factors influencing drug utilization, such as patient adherence, attitudes of healthcare providers, and factors of non‐adherence to the recommended guidelines.

## 6 Conclusion

This study reflects a substantial deviation from WHO prescribing indicators in Pakistan and Yemen, highlighting the immediate need for targeted intervention to promote rational prescribing practices. The alarming rates of polypharmacy, overutilization of antibiotics, and suboptimal generic prescribing not only undermine patient safety but also exacerbate the escalating risk of antimicrobial resistance. To confront these challenges, a multifaceted approach involving education, policy reforms, and an enhanced regulatory framework is required in both countries. Integration of pharmacists into healthcare settings is necessary to improve medication management and promote rational prescribing.

## Author Contributions

**Amjad Khan:** conceptualization, data curation, formal analysis, investigation, methodology, project administration, resources, software, supervision, writing – original draft. **Ali Mohammed Ahmed Al‐Subari:** conceptualization, data curation, investigation, methodology, writing – original draft. **Saima Mushtaq:** conceptualization, data curation, formal analysis, software, writing – review and editing. **Kiran Hameed:** conceptualization, data curation, formal analysis, writing – review and editing. **Sameen Abbas:** conceptualization, data curation, formal analysis, writing – review and editing. **Muhammad Iqbal:** conceptualization, data curation, formal analysis, project administration, writing – review and editing. **Yalin Dong:** conceptualization, supervision, writing – review and editing. **Weiyi Feng:** conceptualization, project administration, supervision, writing – review and editing. **Yu Fang:** conceptualization, project administration, supervision, writing – review and editing.

## Ethics Statement

The bioethics committee of Quaid‐i‐Azam University Islamabad granted ethical approval for the study under study protocol #BEC‐FBS‐QAU2023‐507. Written informed consent was obtained from each participant.

## Conflicts of Interest

The authors declare no conflicts of interest.

## Data Availability

The original contributions presented in the study are included in the article; further inquiries can be directed to the corresponding author.
